# Salt-Resilient Cowpeas: Early Identification Through Growth Parameters and Gene Expression at Germination Stage

**DOI:** 10.3390/ijms26051892

**Published:** 2025-02-22

**Authors:** Patrícia Afonso, Isaura Castro, Márcia Carvalho

**Affiliations:** 1 Centre for the Research and Technology of Agro-Environmental and Biological Sciences (CITAB), University of Trás-os-Montes e Alto Douro (UTAD), 5000-801 Vila Real, Portugal; pafonso@utad.pt (P.A.); icastro@utad.pt (I.C.); 2 Institute for Innovation, Capacity Building and Sustainability of Agri-Food Production (Inov4Agro), University of Trás-os-Montes e Alto Douro (UTAD), 5000-801 Vila Real, Portugal

**Keywords:** *Vigna unguiculata* L. Walp., abiotic stress, growth measurements, germination, lipidic peroxidation, gene expression

## Abstract

Soil salinity is one of the most severe impacts of climate change, negatively affecting plant growth and development. Seed germination and seedling emergence are among the most critical stages susceptible to salt stress, making it important to explore them to identify the most resilient accessions for crop yield improvement. Cowpea (*Vigna unguiculata* L. Walp.) is an important crop due to its ability to fix atmospheric nitrogen, improving soil health, and its high protein content. The main objectives of this study were to screen salt-resilient cowpea accessions from a worldwide collection and to evaluate cowpea responses to salt stress at germination stage through gene expression analysis. A total of 40 cowpea accessions from sixteen different countries were subjected to two treatments: control (water) and salt stress (150 mM NaCl solution). The seeds germinated, and the seedlings grew for ten days. The germination and growth parameters and lipid peroxidation quantification were determined. The results revealed significant differences in all parameters among accessions and treatments. A high variation in salt responses was detected among accessions, allowing the selection of five accessions (Co_2, Co_4, Co_21, Co_30, Co_31) as resilient to salt stress at germination stage. Subsequently, two salt stress-related genes (*DREB2* and *VuEXO*) were evaluated through qPCR, revealing genotype-dependent regulation. These results provide valuable insights for the early selection of salt-resilient cowpea accessions, which may be considered for the development of improved and new varieties in the future.

## 1. Introduction

Climate change, food shortages, degradation of soil, and pollution are among the most urgent global concerns. Irik and Birkmaz [1] referred that approximately 19.5% of irrigated lands and 2.1% of dry lands are affected by salt stress. Salt stress is characterized by high concentration of cations (K^+^, Mg^2+^, Ca^2+^, and Na^+^) and anions (NO_3_^−^ HCO_3_^−^, SO_4_^2−^, and Cl^–^) [2] and has a significant impact on seed germination, plant growth, and development, leading to substantial reductions in crop yields worldwide [3]. Soil salinity can increase due to natural processes such as low rainfall, high surface evaporation, rock weathering, or due to anthropogenic factors including poor natural resource management, such as urbanization or agricultural practices [3,4]. Strategies to mitigate the negative impacts of salinity are essential to counteract this issue. Identifying salt-resilient accessions could be the most cost-effective way to counteract the effects of salinity on crops. In climate change scenarios, selecting tolerant accessions for different abiotic stresses during germination will be fundamental for farmers and crop producers to increase crop production [2,5]. Salt stress negatively affects germination and growth parameters in several plant species [1,6], promoting a decrease of germination percentage, germination rate, and root development [1]. The selection of resilient accessions through germination studies is an important step in mitigating the effects of salt stress.

Several studies refer that plants have defense systems against abiotic stresses, allowing them to survive under adverse conditions. Seed germination and seedling growth stages are the most vulnerable stages in a plant’s life cycle to these stresses [1,7]. Nevertheless, salt stress affects all phases of plant growth and alters several metabolic processes, particularly photosynthetic activity reducing the plant’s chance of survival. Germination is hampered by osmotic stress—physical stimulus that triggers many physiological changes at the cellular level [8]—caused by excessive osmotic potential, which restricts water uptake. Additionally, the accumulation of sodium and chloride ions in the seed can lead to harmful ionic effects [9]. Salt stress results in delayed and reduced seed germination, consequently leading to an up-regulation in abscisic acid (ABA) levels [10]. Furthermore, salt stress has been shown to affect the function of two enzymes involved in ethylene production. By counteracting ABA’s effects, ethylene plays a significant role in promoting dormancy release and seed germination in various plant species. Salinity stress also significantly impacts plant carbon and nitrogen metabolism. Additionally, salinity influences energy metabolism and lipid synthesis, disrupts cellular equilibrium and can lead to excessive generation of reactive oxygen species (ROS) [3,7]. Salinity stress interferes with photosynthesis and, at molecular level, affects transcription, amino acids, and protein synthesis.

For a better understanding of salt stress resilience, it is essential to explore the molecular pathways involved in this process. Resilience to salt stress is a complex trait that involves the coordinated activity of several gene families, which play various roles, including regulating water loss through stomata, ion sequestration, metabolic changes, osmotic adaptations, and antioxidative defense mechanisms [7]. In the last few years, numerous studies have been developed to understand the transcriptional mechanisms controlling responses to abiotic stresses, including cold, drought, and high salinity. These studies have identified several stress-responsive genes [11]. Stress-responsive genes are classified into two main types: functional genes, which encode enzymes involved in producing protective metabolites, antioxidative enzymes, and those involved in lipid biosynthesis, among others, and regulatory genes, which play crucial roles in plant survival during stress and recovery, including stress receptors, protein kinases, transcription factors (TFs), and others [12].

Transcription factors are key regulators of gene expression changes that enable plants to respond to abiotic stresses. The *dehydration-responsive element binding* (*DREB*) gene family plays a crucial role in plant stress response pathways, with *DREB2A* and *DREB2B* being strongly induced under drought and salt stress conditions [13,14]. *DREB2* genes, which belong to the ethylene responsive factor (ERF) transcription factor family and are part of the ABA-independent signal transduction pathway, are considered potential candidates for improving plant stress tolerance due to their presence in several plant crops [13,15]. The exocyst complex protein EXO70, composed of eight subunits, may serve as an important regulatory center for endomembrane dynamics in plants [16]. The *EXO70* gene family is involved in various biological processes in plants, including the regulation of polarized growth and development of the pollen tube, reproduction, and nodule development. It also plays a crucial role in plant–pathogen interactions, abiotic stress responses, and plant immunity [17]*. Exo70A1* is required for polar auxin transport in *Arabidopsis*, and mutations in the gene affect auxin distribution, decreased apical dominance, and result in shorter root hairs [17,18].

Studies point out that the world population will increase of 50% by 2050 and consequently will face a huge challenge of increasing food crop production by using a more sustainable agriculture in a climate change scenario [19]. The production and consumption of crops with high protein content, such grain legumes, will be extremely relevant to supporting the demand for affordable and healthy food. Nowadays, Europe is facing a plant protein deficit, making it fundamental to find strategies to increase their production [20].

Cowpea (*Vigna unguiculata* L. Walp.) is a tropical crop grown in arid and semiarid regions that is highly adapted to drought and heat. However, in this type of soil, the salt compounds are generally not eluted by low frequency of rainfall, leading to an accumulation of salt in the soil and consequently increasing the salt stress for cowpea and other crops [21,22]. Cowpea is a promising, short, warm season, and multipurpose grain legume crop, since it can be consumed in the form of leaves, green pods, and immature and dry beans [23,24]. As with other grain legumes, cowpea has the capacity for nitrogen fixation by rhizobium symbiosis, playing an important role in agricultural ecosystems allowing an increase of soil fertility [25,26], consequently making it important for sustainable farming. Beside these characteristics, cowpea dry grains contain 23–33% of protein [25,27] and its production can be a suitable approach to meet the increasing demand of affordable plant-based protein [28]. One way to counteract the high soil salinity is to screen the more salt-resilient accessions in a germplasm collection that is to be delivered to farmers and producers that are facing salt stress. Unfortunately, few studies have been performed in exploring the accessions screening.

This study aimed to identify and evaluate salt-resilient cowpea (*Vigna unguiculata* L. Walp.) accessions at germination stage by screening a global collection of cowpea accessions and analyzing their response to salt stress through growth parameters and gene expression analysis.

## 2. Results

### 2.1. Definition of the Optimal NaCl Concentrations

**Figure 1:**
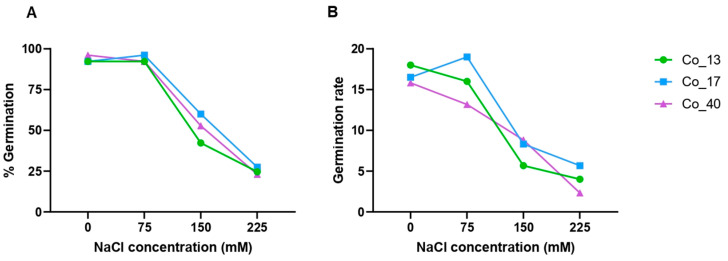
Seed germination percentage (**A**) and rate (**B**) of three cowpea accessions under three salt conditions (75, 150 and 225 mM NaCl concentrations) and control with water (0 mM of NaCl) (n = 3).

A preliminary experiment was performed to determine the most suitable NaCl concentration for screening cowpea accessions resilient to salt stress at germination stage. Four concentrations were selected based on previous studies (0 mM, 75 mM, 150 mM, and 225 mM of NaCl) [2] and are presented in Figure 1.

In the three cowpea accessions, a decrease in the germination percentage and rate with the increasing of NaCl concentrations was observed. The higher concentration (225 mM of NaCl) presented low seed germination percentage and rate, making it difficult to be used for discrimination of the most susceptible accessions. The results of the germination study revealed that salt stress caused a significant decrease in germination rate, particularly at higher NaCl concentrations. Our results showed the concentration of 150 Mm as the most suitable for determining the salt resilience level of a set of 40 cowpea accessions.

### 2.2. Effect of Salt Stress on Germination Parameters

The salt tolerance level of cowpea accessions was initially determined by measuring % germination, germination rate, vigor index, and root and shoot length (Table 1).

The seed germination percentage varied from 73.33% to 100% in both treatments, although the treatment only slightly affected the germination percentage (*p* < 0.05). Significant differences were detected between accessions (*p* < 0.001), which may be related to variations in the germination capacity of each accession at the time of observation (10 days). The germination rate is considered one of the most informative parameters in this type of study. A sharp decrease in germination rate when cowpea seeds were exposed to salt stress (Table 1) was observed, indicating that seeds required more time to germinate under stress conditions. Under control conditions, accessions Co_2 and Co_17 presented the highest germination rate, while under salt stress conditions, accessions Co_9 and Co_17 accessions showed the highest values. Significant differences (*p* < 0.001) were detected between accessions and treatments.

Seedling emergence parameters (root and shoot length) were also affected by salt stress, as root and shoot growth were generally inhibited under salt stress treatment (Table 1). These two parameters presented significant differences (*p* < 0.001) between accessions and root measurements, but only slight differences were presented in shoot measurements (*p* < 0.05). In general, a high impact on root growth under salt stress was observed, while in some accessions, this impact was not observed in relation to shoot growth (e.g., Co_4, Co_16, Co_17, Co_29). Some accessions (Co_1, Co_3, Co_6, Co_7, Co_9, Co_10, Co_11, Co_13, Co_15, Co_18, Co_19, Co_21, Co_22, Co_23, Co_26, Co_27, Co_32, Co_35, Co_38, Co_39) did not produce shoots under salt stress, and accessions Co_30 and Co_31 did not produce shoots under either control or stress conditions, probably due to their sensibility to salt stress.

Germination and seedling emergence parameters may influence plant vigor and, consequently, crop yields. Therefore, it is also important to determine the vigor index (Table 1). It was observed that there was not only a high decrease on the vigor index on cowpea seeds with salt treatment (*p* < 0.001), but also a huge variation between accessions (*p* < 0.001). For this parameter, the Co_20 and Co_25 accessions showed the highest values under control conditions, while the Co_24 and Co_25 accessions exhibited the highest values under salt stress conditions.

The interaction between accession × treatment had a large effect on germination rate, root length (*p* < 0.001), and shoot length (*p* < 0.05). However, there were no significant differences in % germination and vigor index.

### 2.3. Effect of Salt Stress on Lipid Peroxidation Through Malondialdehyde (MDA) Quantification

**Figure 2:**
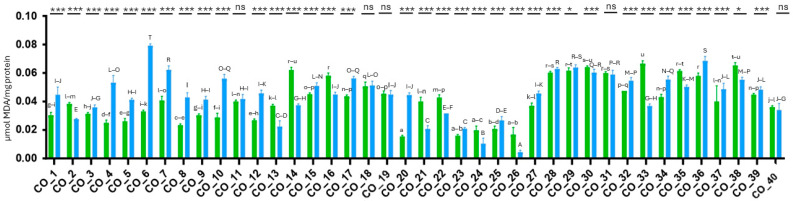
Lipidic peroxidation by MDA quantification evaluated in 40 cowpea accessions under control (control) and salt stress (green) treatments. Means (n = 9) were analyzed with one-way ANOVA followed by Tukey’s test (significance level of 0.05) to compare the accessions, and with one-way ANOVA followed by LSD test to compare treatments (lowercase letters—control treatment; uppercase letters—stress treatment; *—significant differences at level *p* < 0.05; ***—significant differences at level *p* < 0.001; ns—no significant differences).

Lipidic peroxidation through malondialdehyde (MDA) quantification was chosen to determine if seeds were under salt stress or not. Salt stress affected the concentration of MDA in most of the accessions, suggesting that they were under stress (Figure 2). A total of 24 cowpea accessions revealed significant differences (*p* < 0.001) between control and stress treatment. However, 15 cowpea accessions (Co_2, Co_13, Co_14, Co_16, Co_19, Co_21, Co_22, Co_24, Co_26, Co_30, Co_31, Co_33, Co_35, Co_38, Co_40) presented a decrease in the concentration of MDA under salt stress when compared with control treatment, suggesting that these accessions have resilience mechanisms against salt stress. Only five accessions (Co_11, Co_18, Co_19, Co_31, Co_40) did not present significant differences (*p* > 0.05).

### 2.4. Screening of Cowpea Accessions for Salt Stress Resilience

**Figure 3:**
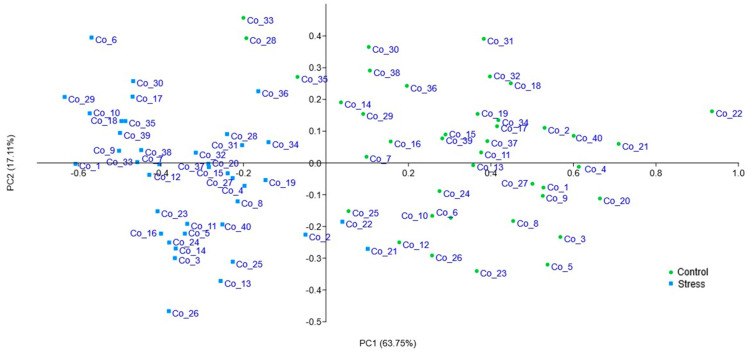
Principal component analysis with the 40 cowpea accessions data obtained in % germination, germination rate, vigor index, and MDA quantification from control and salt stress treatments.

To screen the salt stress resilience and susceptible cowpea accessions, a principal component analysis (PCA; Figure 3) was performed using the growth parameters and MDA normalized data. The principal components explained 80.86% of total variation (PC1 = 63.75% and PC2 = 17.11%), with shoot length (54.9%) and root length (50.8%) being the most contributive parameters for PC1 distribution and the vigor index (52.8%) and MDA quantification (27.4%) for PC2 distribution. From this analysis, the accessions Co_2, Co_18, Co_19, Co_30, and Co_31 were selected as potential salt-resilient accessions, while the Co_10 and Co_4 were selected as salt susceptible accessions. Also, we chose Co_21 accessions, which exhibited an intermediate response pattern.

### 2.5. Gene Expression Profiling to Screen Cowpea Salt Stress Resilience

**Figure 4:**
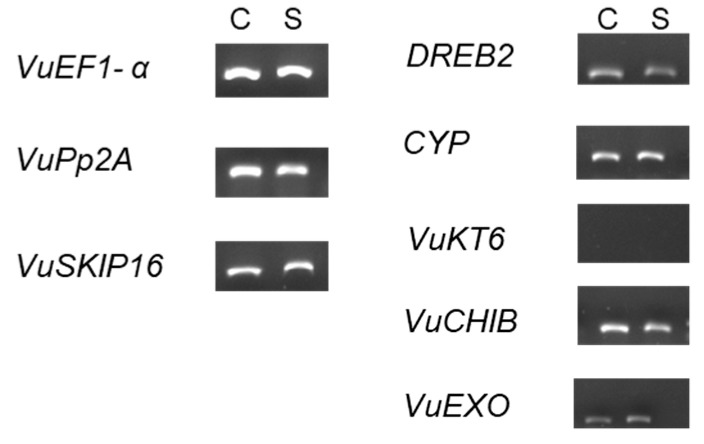
Gene expression profile by semi-quantitative PCR of five salt stress-related genes and three reference genes.

To analyze the gene expression under salt stress, three reference and five salt-related genes with different functions were selected according to the literature, as shown in Table 2. Semi-quantitative RT-PCR was used to analyze their expression profiles and all amplifications revealed a single amplicon (Figure 4).

The analysis software (Image LabTM Software version 1.0, BioRad, Hercules, CA, USA) allowed us to validate the three references and to detect slight differences in the salt-related gene profiles. The *VuEF1-α* and *VuSKIP* genes were selected as reference genes due to their stability under both control and salt stress conditions, while the *DREB2A* and *VuEXO* genes showed slight differences between control and salt stress treatment. For this reason, we considered it important to evaluate them by qPCR and explore these differences. It is important to mention that the *VuKT6* gene did not amplify after several tests, probably due to an error on primer design.

**Figure 5:**
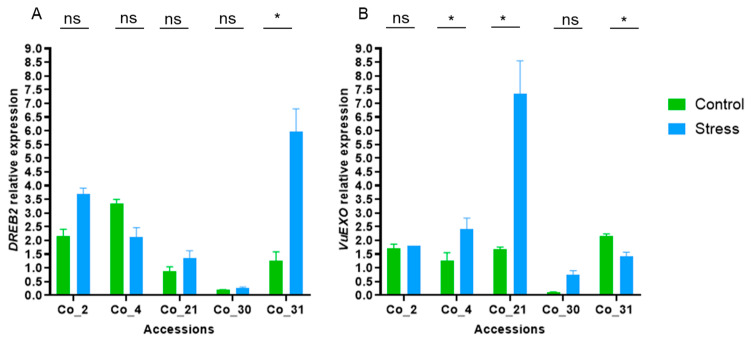
Effect of salt stress treatment in the expression of salt stress-related genes ((**A**)—*DREB2A* and (**B**)—*VuEXO*), as evaluated by qPCR. The relative expression levels were obtained after normalization with the expression of *VuEF1-α* and *VuSkip16* reference genes. Treatments were compared using a T-student test (significance level of 0.05; *—significant differences at level *p* < 0.05; ns—no significant differences).

Gene expression analysis by qPCR from both salt-related genes are presented in Figure 5. In general, an increase in their expression was observed under salt stress treatment, suggesting their association with cowpea salt stress response (Figure 5). Only the accessions Co_4 and Co_31 did not show this increased expression pattern for *DREB2* and *VuEXO* genes, respectively (Figure 5). Under salt conditions, significant overexpression of the *DREB2* gene was observed in accession Co_31, and of the gene *VuEXO* in the accessions Co_4 and Co_21.

## 3. Discussion

Soil salinity is one of the most severe impacts of climate change. This stress negatively affects plant growth and development, resulting in significant losses in global agricultural production. Research has argued that the inhibition or limitation of seed germination by NaCl is caused by the decrease in water uptake due to salt and its toxic effects on the embryo [25]. Seed germination and seedling emergence are among the most critical stages susceptible to salt stress, being therefore particularly important in the selection of tolerant accessions at germination stage [26]. However, it is important to note that the high salt ion concentrations will also affect plant development throughout its life cycle, including a decrease in height, biomass, chlorophyll content, and grain production [22,32].

In vitro screening methods on seeds [5] allow a rapid, accurate, and high-throughput analysis of tolerance at an early growth stage. The use of NaCl to artificially impose salt stress on seeds was crucial for evaluating a total of 40 worldwide cowpea accessions. Different NaCl concentrations were tested (Figure 1) based on the study conducted by Ravelombola et al. [2], and a concentration of 150 mM was determined to be optimal for inducing salt stress, ensuring evident salt stress effects without causing excessive damage to the seedlings. In common bean, 150 mM NaCl concentration was also considered optimal for germination studies [33]. However, other studies on salt stress in cowpea used lower NaCl concentrations (less than 100 mM), including those by Nunes et al. [34] and Tavares et al. [35]. Our experiment was designed to ensure highly controlled growth conditions, consistently using the same incubator and environmental settings (temperature and humidity). Replicates were performed randomly to minimize errors and experimental limitations. The duration of salt stress exposure is a key factor that could influence the results, but for an experiment at germination stage, a ten-day period was chosen based on previous studies [2,36]. Germination percentage and germination rate were the two key parameters used to assess the effect of salt stress on plant germination. The germination percentage (% germination) varied significantly between accessions. However, differences between treatments were smaller in % germination (*p* < 0.05), showing only a slight decrease under salt stress conditions. In both control and salt stress treatments, the % germination varied from 73.33% to 100%, agreeing with findings in other studies [2,34,35]. Notably, the Co_12, Co_16, and Co_31 accessions were the only ones where the % germination was lower in control treatment compared to the salt stress treatment. These results may be attributed to variations in the germination capacity of each accession and the time of the experiment (10 days). The germination rate is considered one of the most informative parameters in this type of study, as it provides insights into the behavior of accessions under salt stress conditions [2,5]. In our study, the germination rate decreased substantially under salt stress conditions compared to the control, with significant differences observed among accessions and treatments. The results demonstrated that, despite being exposed to salt stress, the seeds were able to germinate. However, their germination rate was negatively affected. This suggests that salt stress reduces the germination rate in cowpea, which is consistent with previous studies [2,34]. We also identified significant differences in germination rates among cowpea accessions, indicating that our cowpea accession collection exhibits variability in responses to salt stress, aligning with data reported in the literature [2,6,37]. 

To analyze seed development, it is important to evaluate seedlings growth, specifically root and shoot length, as well as the vigor index. In general, the accessions under study displayed different behaviors under salt stress conditions. Regarding root and shoot length, significant differences were observed across accessions, treatments, and accession–treatment interaction. Other studies [2,34,35] have reported similar findings, showing a tendency for a decrease in seedling growth parameters under salt treatments. In fact, under salt stress, several accessions did not show shoot development. According to the literature, excessive salinity inhibits root and shoot growth by increasing osmotic pressure in the medium, causing ionic toxicity to the roots, and leading to nutritional imbalances in the plant. These imbalances are associated with deficits in nutrient absorption and/or transportation, as well as reduced water uptake by the plant [37,38,39]. The seedling vigor index is another important parameter, as it combines seed germination percentage and seedling growth data [5]. In our study, a decrease in vigor index values was observed under salt stress conditions compared to the control. Significant differences were detected between accessions and treatments, although no differences were observed in the accession-treatment interaction.

When plants are exposed to oxidative stress, they produce H_2_O_2_, which is involved in membrane lipid peroxidation [32]. The generation of malondialdehyde (MDA) is, therefore, considered a marker for stress in plants [32,40]. This compound can be a good stress indicator: a high content of MDA indicates the more sensitive the plant is to stress, while conversely low MDA content in the cell indicates that the plant is more stress-tolerant. Therefore, in some cases, the MDA content has been widely used as an indicator of the tolerance level of the plant to stress, including salt stress [35,39]. Our results showed that salt stress affected MDA concentrations in most of the cowpea accessions, revealing an increase in MDA values. These results are in agreement with other studies developed on cowpea [7]. Studies indicate that MDA concentrations tend to be higher when plants are exposed to stress conditions [29,40]. In our study, 24 accessions exhibited higher MDA concentrations under salt stress compared to the control, while five accessions showed no significant differences. Lower MDA concentrations under salt stress are associated with reduced oxidative damage to membranes and are correlated with stress-resilient behavior in accessions [41,42]. However, Tavares et al. [35] did not observe significative differences in MDA content between cowpea seeds treated with NaCl and the control. We suggest that the use of lower NaCl concentrations (0, 25, 50, 75, and 100 mM) to impose salt stress could explain the absence of significant effects on lipid peroxidation.

To explore the effect of salt stress on cowpea accessions, a gene expression study was conducted. Several genes were tested (Figure 3), and slight differences in gene profiling were detected in the salt stress and control treatments using semi-quantitative RT-PCR. Based on RT-PCR gene profiling, two salt-related genes, *DREB2A* and *VuEXO*, were selected for further expression analysis (Figure 4). Both genes have been described as being involved in abiotic stress responses and were up-regulated when exposed to salt stress conditions [21,40,43]. *DREB2A* is a dehydration-responsive element-binding protein and a transcription factor involved in the regulation of stress-responsive genes, playing a crucial role in osmotic adjustment and cellular protection under salt stress [14]. Meanwhile, *VuEXO,* an exocyst complex protein *EXO70,* is associated with ion homeostasis and cell remodeling; its main function is the central regulation of the endomembrane dynamics in plants, especially when under salt stress [17]. Until now, the exact function of the exocyst gene family had not been identified [21,44], with its newfound importance potentially leading to new avenues for utilization. In general, and in agreement with the literature [21,25], an increase in gene expression was observed for both genes under salt stress treatment compared to control. Exceptions were noted for the *DREB2A* gene in the Co_4 accession and the *VuEXO* gene in the Co_31 accession, which demonstrates cowpeas’ germplasm diversity and genotype-dependency in responses to salt stress. These variations in gene expression between accessions under abiotic stress have been described in the literature as being directly related to the component of genetic diversity and environmental adaptation. It will be crucial in the future to correlate these expression differences with specific genetic loci or phenotypic traits and salt stress resilience [45,46].

In conclusion, this study is an important step towards the selection of cowpea accessions well-adapted to salt stress and, consequently, will contribute to improving crop production. Our findings revealed that germination rate and shoot and root length are the most informative parameters to infer about cowpea accessions and salt stress resilience. The results suggest that Co_2 (Greece), Co_18 (Portugal), Co_19 (Portugal), Co_30 (China), and Co_31 (Benin) accessions have a potential salt resilience at germination stage, while Co_10 (India) and Co_4 (Bulgaria) accessions can be considered the most susceptible. These accessions can be recommended and supplied to farmers with fields facing salinity problems through the collaboration with farmers’ associations or governmental entities. In the future, these accessions can be used as parents for developing new varieties. Beyond the importance of developing accessions better adapted to saline conditions, introducing cowpea into these fields can also enhance soil fertility through its symbiotic relationship with rhizobia, contributing to a more sustainable agricultural system. It will be important to explore in greater depth cowpeas transcriptome and metabolome in salt-stress conditions in both controlled indoor environment and field conditions.

## 4. Materials and Methods

### 4.1. Plant Material

A total of 40 cowpea (*Vigna unguiculata* L. Walp.) accessions were used in this study for salt tolerance evaluation at germination stage (Table 3), with 18 being from the Iberian Peninsula and 22 originally collected in 15 different countries.

### 4.2. Determination of Optimal NaCl Concentration

To test the most suitable NaCl concentration for salt stress germination studies, three cowpea accessions (Portuguese variety ‘Fradel’, advanced breeding line IT97K-499-95 and landrace Co_17) were submitted to three different levels of stress (75 mM, 150 mM, and 225 mM). Distilled water (without NaCl) was used as control. Germination assays were performed in an incubator (Binder Incubator series D, Germany) in the dark during 10 days at 25 ± 1 °C. Uniform seeds from each cowpea accession were selected. To avoid fungal development, the seeds were disinfected in a 10% sodium hypochlorite solution for about 5 min. Then, seeds were washed three times with sterile distilled water for about 5 min. The seeds were grown in a Petri dish with filter paper and sealed with Parafilm to avoid evaporation and contamination. A total of three replicates of each treatment/accession combination were performed. The optimal salt concentration was determined based on the most significant difference in germination rates across cowpea accessions.

### 4.3. Germination Conditions and Experimental Design

Salt stress was induced using a NaCl solution of 150 mM (hereinafter referred as stress). Three replicates of each treatment/accession combination were performed, with a total of 30 seeds per combination. These combinations were placed separately on three different incubator shelves, with each shelf considered as a block. The seeds were grown in the dark for ten days in an incubator with a controlled temperature of 25 ± 1 °C. The roots were collected for measurements and biochemical and molecular analysis, and were kept at −80 °C.

### 4.4. Growth Measurements and Data Collection

Following Ravelombola et al. [2] and Carvalho et al. [5], a seed was considered germinated when the radicle has one-third of seed length. Germination data were recorded daily for ten days. The length of roots and shoots were measured (RL and SL, respectively), and the roots were frozen in liquid nitrogen and kept at −80 °C. The percentage of seed germination (% G), seed germination rate (GR), and vigour index (VI) were calculated according to Carvalho et al. [5]

### 4.5. MDA Determination

The MDA-TBA methodology analyses malondialdehyde (MDA), a product of lipid peroxidation, through its reaction with thiobarbituric acid (TBA).

Following the methodology optimized by Carvalho et al. [40], aliquots from control and salt stress samples were used to determine the lipidic peroxidation by MDA quantification. The supernatant’s absorbance was measured at 532 and 600 nm and the amount of MDA-TBA complex was calculated by subtracting the A_532_ to A_260_ and using the extinction coefficient of 155 mm^−1^ cm^−1^.

### 4.6. Gene Expression of Salt-Related Genes

Pools of root samples from each accession and treatment were grounded into a fine powder with liquid nitrogen, and total RNA was extracted using the NucleoSpin RNA Plant kit (Macherey-Nagel, Düren, Germany), following the manufacturer’s instructions. cDNA was synthesized using the SensiFAST cDNA Synthesis kit (Meridian Bioscience, Memphis, TN, USA) for a total RNA concentration of 1000 ng/μL, following the manufacturer’s procedure.

A total of five salt stress-related and three reference genes (Table 2) were tested by semi-quantitative PCR using the Taq PCR Master mix kit (Qiagen, Hilden, Germany). Specific primers were designed based on sequences available in the NCBI database and/or described in previous studies. The PCR amplifications were carried out in a BIO-RAD T100TM thermal cycler (BioRad, Hercules, USA) and the amplicons were separated by electrophoresis in agarose gels (1.5%, *w*/*v*), at 90 V for 35 min. Gels were visualized using the Molecular Image Gel-DocTM XR^+^ with Image LabTM Software (BioRad, Hercules, CA, USA). Two differentially expressed genes (*DREB2A* and *VuEXO*) were tested using the SensiFAST Sybr HI-ROX mix (Meridian Bioscience, Memphis, TN, USA) and analyzed with the StepOnePlus Real Time PCR system (Applied Biosystems, Foster City, CA, USA), using relative quantification according to the MIQE criteria (Minimum Information for the Publication of Quantitative Real-Time PCR Experiments; [47]) and the previous studies [40].

The amplifications were performed in triplicate and evaluated with the StepOnePlus Real Time PCR software (Applied Biosystems, Foster City, CA, USA). The mean PCR efficiency per gene was calculated using standard curves based on five-fold dilutions of the cDNA pool from all samples (in triplicate). The reference and target genes’ efficiency levels ranged from 90% to 115%. *VuEF1-α* and *VuSKIP* were utilized as reference genes for normalization. The relative quantification per gene was calculated according to the 2^−ΔΔCt^ method, where ΔCt is the difference in threshold cycle between the average mean of the target and average of the two reference genes, and ΔΔCt is the difference between the average ΔCt of the target and control samples.

### 4.7. Data Analysis

Data from germination (% G, GR, and VI) are presented as the mean of three independent assays (n = 3). Growth measurements (root and shoot length) and lipidic peroxidation by MDA content are presented as the mean of 9 repetitions (n = 9). Differences between means were analyzed with one- or two-way ANOVA and Tukey’s test (*p* < 0.05 is considered significant) using IBM SPSS Statistics software version 20 (IBM SPSS, Inc., Chicago, IL, USA). All graphics were created with GraphPad Prism (version 8.0.0).

Principal component analysis (PCA) was performed using Past statistical software version 3.19 [48] using normalized values. The normalization was performed for percentages, taking into consideration the highest value achieved for each parameter.

## Figures and Tables

**Table 1:** Effect of salinity on germination and seedling growth parameters. Significant differences were evaluated by one-way or two-way ANOVA, followed by Tukey tests at *p* < 0.05 (lowercase letters—control treatment; uppercase letters—stress treatment; dark gray—significant differences at level *p* < 0.001; gray—significant differences at level *p* < 0.01; light gray—significant differences at level *p* < 0.05; white/clear—no significant differences).

Accession	Treatment	%Germination	Germination Rate	Root Length	Shoot Length	Vigour Index
Co_1	Control	100.00 ± 0.00 ^b^	3.61 ± 0.84 ^a–f^	7.86 ± 1.86 ^a–h^	1.68 ± 0.63 ^c–h^	955.67 ± 116.51 ^a,b^
	Salt stress	86.67 ± 11.55 ^A^	2.06 ± 0.26 ^A–C^	2.19 ± 0.68 ^A–E^	without shoot	191.47 ± 44.06 ^A,B^
Co_2	Control	100.00 ± 0.00 ^b^	4.83 ± 0.29 ^e,f^	7.99 ± 2.82 ^b–h^	1.39 ± 0.55 ^b–h^	973.33 ± 143.20 ^a,b^
	Salt stress	100.00 ± 0.00 ^A^	3.06 ± 0.42 ^A–D^	3.57 ± 0.68 ^D–J^	1.11 ± 0.49 ^A,B^	468.33 ± 22.19 ^D–F^
Co_3	Control	73.33 ± 11.55 ^a^	2.42 ± 1.02 ^a,b^	9.32 ± 3.36 ^f–h^	2.17 ± 0.50 ^g–h^	843.33 ± 135.77 ^a,b^
	Salt stress	73.33 ± 11.55 ^A^	2.17 ± 0.29 ^A–D^	1.98 ± 0.54 ^A^	without shoot	165.07 ± 13.15 ^A^
Co_4	Control	100.00 ± 0.00 ^b^	4.50 ± 0.50 ^d–f^	6.79 ± 2.85 ^d–h^	1.45 ± 0.46 ^b–h^	1009.50 ± 154.11 ^a,b^
	Salt stress	86.67 ± 11.55 ^A^	2.36 ± 0.97 ^A–D^	3.08 ± 1.02 ^A–J^	1.20 ± 0.25 ^A,B^	370.50 ± 78.78 ^A–F^
Co_5	Control	80.00 ± 0.00 ^a,b^	4.17 ± 0.76 ^b–f^	6.75 ± 2.82 ^a–g^	2.29 ± 0.74 ^h^	648.67 ± 349.84 ^a^
	Salt stress	80.00 ± 0.00 ^A^	2.17 ± 0.44 ^A–D^	2.14 ± 0.63 ^A–C^	1.00 ± 0.00 ^A,B^	224.67 ± 63.89 ^A–C^
Co_6	Control	86.67 ± 11.55 ^a,b^	3.67 ± 0.29 ^a–f^	5.92 ± 1.64 ^a–e^	1.67 ± 0.35 ^c–h^	653.33 ± 148.44 ^a^
	Salt stress	86.67 ± 11.55 ^A^	2.31 ± 0.21 ^A–D^	3.29 ± 1.09 ^A–J^	without shoot	281.33 ± 73.79 ^A–D^
Co_7	Control	100.00 ± 0.00 ^b^	2.44 ± 0.10 ^a,b^	6.70 ± 1.68 ^a–g^	0.33 ± 0.58 ^a^	703.33 ± 170.39 ^a,b^
	Salt stress	100.00 ± 0.00 ^A^	1.86 ± 0.41 ^A–C^	2.00 ± 0.54 ^A^	without shoot	233.33 ± 70.95 ^A–D^
Co_8	Control	93.33 ± 11.55 ^a,b^	4.17 ± 0.58 ^b–f^	6.87 ± 2.79 ^a–g^	1.64 ± 0.69 ^c–h^	783.56 ± 130.48 ^a,b^
	Salt stress	93.33 ± 11.55 ^A^	2.61 ± 0.19 ^A–D^	3.07 ± 0.70 ^A–J^	1.00 ± 0.00 ^A,B^	364.83 ± 46.69 ^A–F^
Co_9	Control	100.00 ± 0.00 ^b^	4.33 ± 0.76 ^c–f^	6.90 ± 1.51 ^a–g^	1.78 ± 0.57 ^d–h^	874.72 ± 119.65 ^a,b^
	Salt stress	100.00 ± 0.00 ^A^	3.22 ± 0.35 ^B–D^	2.15 ± 0.69 ^A–D^	without shoot	215.33 ± 34.43 ^A,B^
Co_10	Control	100.00 ± 0.00 ^b^	4.67 ± 0.29 ^d–f^	4.53 ± 0.61 ^a^	1.50 ± 0.45 ^b–h^	590.00 ± 43.59 ^a^
	Salt stress	100.00 ± 0.00 ^A^	2.44 ± 0.10 ^A–D^	2.33 ± 0.56 ^A–G^	without shoot	233.33 ± 25.17 ^A–D^
Co_11	Control	100.00 ± 0.00 ^b^	3.06 ± 0.42 ^a–e^	7.77 ± 2.50 ^a–h^	1.40 ± 0.66 ^b–h^	908.33 ± 185.09 ^a,b^
	Salt stress	100.00 ± 0.00 ^A^	2.06 ± 0.10 ^A–C^	2.13 ± 0.58 ^A–C^	without shoot	246.67 ± 40.42 ^A–D^
Co_12	Control	93.33 ± 11.55 ^a,b^	3.00 ± 0.00 ^a–d^	5.17 ± 1.67 ^a–c^	1.50 ± 0.35 ^b–h^	618.00 ± 43.24 ^a^
	Salt stress	100.00 ± 0.00 ^A^	2.36 ± 0.13 ^A–D^	2.37 ± 0.95 ^A–H^	0.50 ± 0.00 ^A^	253.33 ± 72.34 ^A–D^
Co_13	Control	93.33 ± 11.55 ^a,b^	3.67 ± 1.04 ^a–f^	4.86 ± 1.67 ^a–b^	1.56 ± 0.90 ^b–h^	599.00 ± 150.74 ^a^
	Salt stress	73.33 ± 30.55 ^A^	1.72 ± 0.84 ^A–C^	2.27 ± 1.13 ^A–F^	without shoot	172.33 ± 61.04 ^A^
Co_14	Control	100.00 ± 0.00 ^b^	4.50 ± 0.00 ^d–f^	6.13 ± 1.59 ^a–f^	1.43 ± 0.50 ^b–h^	756.67 ± 148.44 ^a,b^
	Salt stress	86.67 ± 23.09 ^A^	2.56 ± 0.75 ^A–D^	2.33 ± 0.81 ^A–G^	1.00 ± 0.00 ^A,B^	248.61 ± 96.94 ^A–D^
Co_15	Control	93.33 ± 11.55 ^a,b^	3.33 ± 0.58 ^a–f^	5.43 ± 1.47 ^a–d^	1.10 ± 0.22 ^a–f^	588.17 ± 241.90 ^a^
	Salt stress	86.67 ± 23.09 ^A^	1.83 ± 0.58 ^A–C^	2.07 ± 0.83 ^A–B^	without shoot	216.00 ± 116.83 ^A,B^
Co_16	Control	86.67 ± 23.09 ^a,b^	4.50 ± 0.87 ^d–f^	6.67 ± 1.32 ^a–g^	1.25 ± 0.26 ^b–g^	688.33 ± 199.77 ^a,b^
	Salt stress	100.00 ± 0.00 ^A^	3.11 ± 0.67 ^A–D^	2.57 ± 0.78 ^A–I^	1.00 ± 0.00 ^A,B^	290.00 ± 121.44 ^A–E^
Co_17	Control	100.00 ± 0.00 ^b^	4.83 ± 0.29 ^e,f^	6.75 ± 1.89 ^a–g^	1.23 ± 0.26 ^a–g^	810.28 ± 151.44 ^a,b^
	Salt stress	93.33 ± 11.55 ^A^	3.83 ± 1.04 ^D^	2.96 ± 0.75 ^A–J^	1.00 ± 0.00 ^a,b^	313.17 ± 135.81 ^A–E^
Co_18	Control	100.00 ± 0.00 ^b^	5.00 ± 0.00 ^f^	6.70 ± 1.72 ^a–g^	1.38 ± 0.31 ^b–h^	807.78 ± 56.99 ^a,b^
	Salt stress	93.33 ± 11.55 ^A^	3.17 ± 0.29 ^B–D^	3.07 ± 0.92 ^A–J^	without shoot	276.50 ± 37.37 ^A–D^
Co_19	Control	100.00 ± 0.00 ^b^	4.67 ± 0.29 ^d–f^	7.97 ± 2.94 ^b–h^	1.23 ± 0.50 ^a–g^	920.00 ± 270.74 ^a,b^
	Salt stress	86.67 ± 23.09 ^A^	2.94 ± 0.82 ^A–D^	2.89 ± 0.79 ^A–J^	without shoot	251.83 ± 72.87 ^A–D^
Co_20	Control	100.00 ± 0.00 ^b^	3.67 ± 0.29 ^a–f^	9.67 ± 2.30 ^g–h^	1.43 ± 0.29 ^b–h^	1112.22 ± 173.33 ^a,b^
	Salt stress	100.00 ± 0.00 ^A^	2.39 ± 0.19 ^A–D^	3.63 ± 1.11 ^F–K^	0.58 ± 0.46 ^A,B^	400.00 ± 52.92 ^A–F^
Co_21	Control	100.00 ± 0.00 ^b^	2.94 ± 0.82 ^a–d^	5.64 ± 1.73 ^a–e^	0.79 ± 0.39 ^a–c^	656.94 ± 30.42 ^a^
	Salt stress	86.67 ± 11.55 ^A^	2.33 ± 0.29 ^A–D^	3.07 ± 0.92 ^A–J^	without shoot	295.33 ± 35.01 ^A–E^
Co_22	Control	100.00 ± 0.00 ^b^	2.56 ± 0.42 ^a–c^	8.13 ± 2.94 ^b–h^	0.90 ± 0.42 ^a–d^	905.00 ± 118.22 ^a,b^
	Salt stress	100.00 ± 0.00 ^A^	1.78 ± 0.10 ^A–C^	4.03 ± 1.14 ^J–K^	without shoot	403.33 ± 20.82 ^A–F^
Co_23	Control	93.33 ± 11.55 ^a,b^	2.50 ± 0.50 ^a,b^	6.75 ± 2.31 ^a–g^	1.70 ± 0.45 ^c–h^	812.17 ± 285.61 ^a,b^
	Salt stress	93.33 ± 11.55 ^A^	2.44 ± 0.10 ^A–D^	3.50 ± 0.93 ^C–J^	without shoot	332.67 ± 108.08 ^A–E^
Co_24	Control	100.00 ± 0.00 ^b^	4.50 ± 0.50 ^d–f^	8.68 ± 3.88 ^d–h^	1.89 ± 0.45 ^e–h^	1065.83 ± 105.49 ^a,b^
	Salt stress	93.33 ± 11.55 ^a^	3.00 ± 0.50 ^A–D^	5.00 ± 1.23 ^K^	1.23 ± 0.26 ^A,B^	580.17 ± 76.11 ^F^
Co_25	Control	100.00 ± 0.00 ^b^	4.67 ± 0.58 ^d–f^	10.73 ± 3.23 ^h^	2.04 ± 0.54 ^f–h^	1280.00 ± 298.66 ^b^
	Salt stress	100.00 ± 0.00 ^A^	3.28 ± 0.10 ^C–D^	3.97 ± 1.48 ^I–K^	1.27 ± 0.33 ^B^	524.17 ± 13.77 ^E–F^
Co_26	Control	100.00 ± 0.00 ^b^	3.11 ± 0.54 ^a–e^	5.70 ± 2.17 ^a–e^	1.44 ± 0.32 ^b–h^	713.06 ± 106.39 ^a,b^
	Salt stress	100.00 ± 0.00 ^A^	2.22 ± 0.10 ^A–D^	2.08 ± 0.90 ^A–C^	without shoot	224.67 ± 74.87 ^A–C^
Co_27	Control	100.00 ± 0.00 ^b^	2.94 ± 0.42 ^a–d^	5.43 ± 2.42 ^a–d^	1.30 ± 0.45 ^b–g^	665.83 ± 225.89 ^a,b^
	Salt stress	100.00 ± 0.00 ^A^	2.11 ± 0.10 ^A–C^	2.10 ± 0.99 ^A–C^	without shoot	210.00 ± 40.00 ^A,B^
Co_28	Control	86.67 ± 23.09 ^a,b^	3.17 ± 0.76 ^a–e^	8.29 ± 3.09 ^c–h^	1.77 ± 0.41 ^d–h^	908.89 ± 336.01 ^a,b^
	Salt stress	86.67 ± 11.55 ^A^	3.17 ± 0.76 ^A–D^	3.19 ± 1.55 ^A–J^	0.83 ± 0.35 ^A,B^	320.00 ± 150.997 ^A–E^
Co_29	Control	100.00 ± 0.00 ^b^	4.67 ± 0.29 ^d–f^	8.17 ± 2.83 ^b–h^	1.10 ± 0.22 ^a–f^	919.444 ± 121.864 ^a,b^
	Salt stress	86.67 ± 23.09 ^A^	3.00 ± 1.32 ^A–D^	3.05 ± 1.30 ^A–J^	1.00 ± 0.25 ^A,B^	345.833 ± 177.056 ^A–F^
Co_30	Control	100.00 ± 0.00 ^b^	2.22 ± 0.26 ^a^	6.97 ± 1.92 ^a–g^	without shoot	696.667 ± 134.288 ^a,b^
	Salt stress	100.00 ± 0.00 ^A^	2.17 ± 0.00 ^A–D^	3.43 ± 0.88 ^B–J^	without shoot	343.333 ± 30.551 ^A–F^
Co_31	Control	93.33 ± 11.55 ^a,b^	3.33 ± 0.58 ^a–f^	6.17 ± 1.31 ^a–f^	without shoot	580.000 ± 151.00 ^a^
	Salt stress	100.00 ± 0.00 ^A^	2.50 ± 0.00 ^A–D^	3.33 ± 0.86 ^A–J^	without shoot	366.67 ± 65.06 ^A–F^
Co_32	Control	100.00 ± 0.00 ^b^	3.83 ± 1.26 ^a–f^	6.70 ± 1.87 ^a–g^	0.67 ± 0.29 ^a,b^	725.56 ± 179.33 ^a,b^
	Salt stress	100.00 ± 0.00 ^A^	2.67 ± 0.29 ^A–D^	3.43 ± 0.68 ^B–J^	without shoot	343.33 ± 11.55 ^A–F^
Co_33	Control	100.00 ± 0.000 ^b^	4.67 ± 0.29 ^d–f^	8.17 ± 3.63 ^b–h^	0.97 ± 0.30 ^a–e^	913.33 ± 408.21 ^a,b^
	Salt stress	93.33 ± 11.55 ^A^	2.56 ± 0.42 ^A–D^	3.31 ± 1.00 ^A–J^	0.75 ± 0.30 ^a,b^	321.89 ± 107.77 ^A–E^
Co_34	Control	100.00 ± 0.00 ^b^	4.67 ± 0.58 ^d–f^	7.27 ± 2.03 ^f–k^	1.32 ± 0.33 ^b–g^	855.83 ± 109.61 ^a,b^
	Salt stress	100.00 ± 0.00 ^A^	3.11 ± 0.67 ^A–D^	3.63 ± 1.25 ^F–K^	1.00 ± 0.00 ^A,B^	430.00 ± 43.59 ^B–F^
Co_35	Control	100.00 ± 0.00 ^b^	2.56 ± 0.26 ^a–c^	5.97 ± 2.39 ^a–f^	0.64 ± 0.24 ^a,b^	668.89 ± 267.71 ^a,b^
	Salt stress	100.00 ± 0.00 ^A^	1.78 ± 0.19 ^A–C^	3.73 ± 0.90 ^G–K^	without shoot	373.33 ± 20.82 ^A–F^
Co_36	Control	100.00 ± 0.00 ^b^	4.44 ± 0.51 ^d–f^	6.70 ± 3.18 ^a–e^	1.10 ± 0.39 ^a–f^	698.33 ± 53.41 ^a,b^
	Salt stress	100.00 ± 0.00 ^A^	3.17 ± 0.17 ^B–D^	3.77 ± 1.37 ^H–K^	0.90 ± 0.21 ^A,B^	465.56 ± 27.96 ^C–F^
Co_37	Control	100.00 ± 0.00 ^b^	2.28 ± 0.10 ^a^	8.97 ± 2.39 ^e–h^	1.33 ± 0.41 ^b–h^	1013.33 ± 217.33 ^a,b^
	Salt stress	100.00 ± 0.00 ^A^	2.06 ± 0.10 ^A–C^	3.60 ± 1.17 ^E–K^	0.75 ± 0.35 ^A,B^	385.00 ± 109.66 ^A–F^
Co_38	Control	100.00 ± 0.00 ^b^	2.22 ± 0.19 ^a^	7.57 ± 1.52 ^a–h^	1.00 ± 0.71 ^a–e^	782.50 ± 126.47 ^a,b^
	Salt stress	93.33 ± 11.55 ^A^	1.44 ± 0.19 ^A^	3.23 ± 0.93 ^A–J^	without shoot	323.17 ± 96.94 ^A–E^
Co_39	Control	93.33 ± 11.55 ^a,b^	3.17 ± 0.29 ^a–e^	7.47 ± 1.51 ^a–h^	1.25 ± 0.42 ^g–h^	810.11 ± 131.30 ^a,b^
	Salt stress	93.33 ± 11.55 ^A^	1.56 ± 0.19 ^A,B^	3.86 ± 1.05 ^I–K^	without shoot	364.00 ± 75.02 ^A–F^
Co_40	Control	100.00 ± 0.00 ^b^	4.67 ± 0.29 ^d–f^	8.50 ± 1.89 ^c–h^	1.50 ± 0.62 ^b–h^	1002.78 ± 119.89 ^a,b^
	Salt stress	86.67 ± 11.55 ^A^	2.53 ± 0.17 ^A–D^	3.04 ± 0.77 ^A–J^	0.83 ± 0.26 ^A,B^	335.67 ± 38.55 ^A–E^
*p-*value (accession)		<0.001	<0.001	<0.001	<0.001	<0.001
*p-*value (treatment)		0.011	<0.001	<0.001	<0.001	<0.001
*p-*value (accession × treatment)		0.858	<0.001	<0.001	0.020	0.074

**Table 2:** Salt-related and reference genes tested by semi-quantitative PCR. (Fs—fragment size; Ta—annealing temperature).

Gene Type	Gene	Gene Function	Primer Sequence (5′3′)	Fs (bp)	Ta (°C)	Reference
Reference	*VuEF1-α*	Elongation factor 1-alpha	F: GCCTGGTATGGTGGTGACTT R: GCGAACTTCACTGCAATGTG	280	60	[29]
	*VuPp2A*	Regulatory subunit of phosphatase 2A protein	F: CATTGTTGAGCTTGCTGAGG R: GAGCACCAAGCTTGTCATCA	150	60	[30]
	*VuSkip16*	ASK-interacting protein 16	F: ACAGCCGTGAACAAAAAGG R: GTGGCTTCTTCGTCCACACT	300	60	[29]
Salt stress related	*Cyp*	Plant protein	F: TGCCCGGAAATCAGTTTTGG R: TCAATAAAGGCCGCATGGTG	100	60	[21]
	*VuKT6*	Gene encoding potassium transporter 6	F: GGTAATGCCTCAGGTTTGGC R: CGAGTGCAAGGAGGACATTC	106	60	[21]
	*VuEXO*	Exocyst complex protein EXO70	F: GTCAAAGACGTGGAAGGCTG R: GTTGTCGGCTGTTATGGTGG	175	60	[21]
	*VuCHiB*	Hydrolytic enzyme chitinase	F: GGTAATGCCTCAGGTTTGGC R: CGAGTGCAAGGAGGACATTC	130	58	[31]
	*DREB2A*	Transcription factor gene	F: TTTGTGGACATGGGTGCTTA R: TCCCTTTCATGCATCCTTTC	150	60	This study

**Table 3:** Cowpea accessions used, origin and current status.

**Accession Code**	**Origin Country**	Material Type *
Co_1	Greece	Landrace
Co_2	Greece	Landrace
Co_3	Colombia	Landrace
Co_4	Bulgaria	Landrace
Co_5	Brazil	Landrace
Co_6	Portugal	Landrace
Co_7	Spain	Landrace
Co_8	Iran	Landrace
Co_9	China	Landrace
Co_10	India	Landrace
Co_11	Italy	Landrace
Co_12	Brazil	Landrace
Co_13	Portugal	Variety ^a^
Co_14	Portugal	Landrace
Co_15	Portugal	Landrace
Co_16	Portugal	Landrace
Co_17	Portugal	Landrace
Co_18	Portugal	Landrace
Co_19	Portugal	Landrace
Co_20	Italy	Landrace
Co_21	Spain	Landrace
Co_22	Italy	Landrace
Co_23	Spain	Landrace
Co_24	Spain	Landrace
Co_25	Spain	Landrace
Co_26	Spain	Landrace
Co_27	Congo	Landrace
Co_28	Cuba	Landrace
Co_29	Irak	Landrace
Co_30	China	Landrace
Co_31	Benin	Landrace
Co_32	Angola	Landrace
Co_33	Spain	Landrace
Co_34	Uzbekistan	Landrace
Co_35	Spain	Landrace
Co_36	Spain	Landrace
Co_37	China	Landrace
Co_38	Spain	Landrace
Co_39	Iran	Landrace
Co_40	Nigeria	Advanced breeding line ^b^

* Material type common name: a variety Fradel; b advanced line IT97K-499-35.

## Data Availability

The original contributions presented in this study are included in the article. Further inquiries can be directed to the corresponding author(s).

## References

[1] Irik H.A., Bikmaz G. (2024). Effect of Different Salinity on Seed Germination, Growth Parameters and Biochemical Contents of Pumpkin (*Cucurbita pepo* L.) Seeds Cultivars. Sci. Rep..

[2] Ravelombola W.S., Shi A., Weng Y., Clark J., Motes D., Chen P., Srivastava V. (2017). Evaluation of Salt Tolerance at Germination Stage in Cowpea [*Vigna unguiculata* (L.) Walp]. HortScience.

[3] Hameed A., Ahmed M.Z., Hussain T., Aziz I., Ahmad N., Gul B., Nielsen B.L. (2021). Effects of Salinity Stress on Chloroplast Structure and Function. Cells.

[4] Pereira E.D., Marinho A.B., Ramos E.G., Fernandes C.N.D., Borges F.R.M., de Nazaré José Adriano J. (2019). Saline Stress Effect on Cowpea Beans Growth under Biofertilizer Correction. Biosci. J..

[5] Carvalho M., Matos M., Castro I., Monteiro E., Rosa E., Lino-Neto T., Carnide V. (2019). Screening of Worldwide Cowpea Collection to Drought Tolerant at a Germination Stage. Sci. Hortic..

[6] Zahedi S.M., Ansari A., Azizi M. (2012). The Study of the Effect of Salinity Stress on the Germination and the Initial Growth of Cowpea (*Vigna unguiculata* L. Walp). J. Agric. Technol..

[7] Praxedes S.C., Damatta F.M., De Lacerda C.F., Prisco J.T., Gomes-Filho E. (2014). Salt Stress Tolerance in Cowpea Is Poorly Related to the Ability to Cope with Oxidative Stress. Acta Bot. Croat..

[8] Yu B., Chao D.Y., Zhao Y. (2024). How Plants Sense and Respond to Osmotic Stress. J. Integr. Plant Biol..

[9] Begum M.A. (2013). Saline Stress on Seed Germination. Sci. Res. Essays.

[10] Shu K., Qi Y., Chen F., Meng Y., Luo X., Shuai H., Zhou W., Ding J., Du J., Liu J. (2017). Salt Stress Represses Soybean Seed Germination by Negatively Regulating GA Biosynthesis While Positively Mediating ABA Biosynthesis. Front. Plant Sci..

[11] Qin F., Kakimoto M., Sakuma Y., Maruyama K., Osakabe Y., Tran L.S.P., Shinozaki K., Yamaguchi-Shinozaki K. (2007). Regulation and Functional Analysis of *ZmDREB2A* in Response to Drought and Heat Stresses in *Zea mays* L. Plant J..

[12] Agarwal P.K., Gupta K., Lopato S., Agarwal P. (2017). Dehydration Responsive Element Binding Transcription Factors and Their Applications for the Engineering of Stress Tolerance. J. Exp. Bot..

[13] Li X., Zhang D., Li H., Wang Y., Zhang Y., Wood A.J. (2014). *EsDREB2B*, a Novel Truncated DREB2-Type Transcription Factor in the Desert Legume *Eremosparton songoricum*, Enhances Tolerance to Multiple Abiotic Stresses in Yeast and Transgenic Tobacco. BMC Plant Biol..

[14] Mizoi J., Shinozaki K., Yamaguchi-Shinozaki K. (2012). AP2/ERF Family Transcription Factors in Plant Abiotic Stress Responses. Biochim. Biophys. Acta-Gene Regul. Mech..

[15] Joshi R., Wani S.H., Singh B., Bohra A., Dar Z.A., Lone A.A., Pareek A., Singla-Pareek S.L. (2016). Transcription Factors and Plants Response to Drought Stress: Current Understanding and Future Directions. Front. Plant Sci..

[16] Žárský V., Kulich I., Fendrych M., Pečenková T. (2013). Exocyst Complexes Multiple Functions in Plant Cells Secretory Pathways. Curr. Opin. Plant Biol..

[17] Liu L., Gu C., Zhang J., Guo J., Zhang X., Zhou Z. (2023). Genome-Wide Analysis of Exocyst Complex Subunit Exo70 Gene Family in Cucumber. Int. J. Mol. Sci..

[18] Zhao J., Zhang X., Wan W., Zhang H., Liu J., Li M., Wang H., Xiao J., Wang X. (2019). Identification and Characterization of the *EXO70* Gene Family in Polyploid Wheat and Related Species. Int. J. Mol. Sci..

[19] Gupta S., Kaur R., Sharma T., Bhardwaj A., Sharma S., Sohal J.S., Singh S.V. (2023). Multi-Omics Approaches for Understanding Stressor-Induced Physiological Changes in Plants: An Updated Overview. Physiol. Mol. Plant Pathol..

[20] van Loon M.P., Alimagham S., Pronk A., Fodor N., Ion V., Kryvoshein O., Kryvobok O., Marrou H., Mihail R., Mínguez M.I. (2023). Grain Legume Production in Europe for Food, Feed and Meat-Substitution. Glob. Food Sec..

[21] Kang B.H., Kim W.J., Chowdhury S., Moon C.Y., Kang S., Kim S.H., Jo S.H., Jun T.H., Do Kim K., Ha B.K. (2023). Transcriptome Analysis of Differentially Expressed Genes Associated with Salt Stress in Cowpea (*Vigna unguiculata* L.) during the Early Vegetative Stage. Int. J. Mol. Sci..

[22] Ravelombola W., Shi A., Huynh B.L., Qin J., Xiong H., Manley A., Dong L., Olaoye D., Bhattarai G., Zia B. (2022). Genetic Architecture of Salt Tolerance in a Multi-Parent Advanced Generation Inter-Cross (MAGIC) Cowpea Population. BMC Genom..

[23] Carvalho M., Carnide V., Sobreira C., Castro I., Coutinho J., Barros A., Rosa E. (2022). Cowpea Immature Pods and Grains Evaluation: An Opportunity for Different Food Sources. Plants.

[24] Boukar O., Belko N., Chamarthi S., Togola A., Batieno J., Owusu E., Haruna M., Diallo S., Umar M.L., Olufajo O. (2019). Cowpea (*Vigna unguiculata*): Genetics, Genomics and Breeding. Plant Breed..

[25] Carvalho M., Lino-Neto T., Rosa E., Carnide V. (2017). Cowpea: A Legume Crop for a Challenging Environment. J. Sci. Food Agric..

[26] Stagnari F., Maggio A., Galieni A., Pisante M. (2017). Multiple Benefits of Legumes for Agriculture Sustainability: An Overview. Chem. Biol. Technol. Agric..

[27] Padillaa E.G., Sáncheza R.C.L., Eichler-Loebermannb B., Fernández-Pascualc M., Katia, Barreroa A., Martíneza L.A. Salt Stress Effects on Cowpea (*Vigna unguiculata* L. Walp.) Varieties at Different Growing Stages. Proceedings of the Conference on International Research on Food Security, Natural Resource Management and Rural Development.

[28] Ma Y., Dias M.C., Freitas H. (2020). Drought and Salinity Stress Responses and Microbe-Induced Tolerance in Plants. Front. Plant Sci..

[29] Weiss J., Terry M.I., Martos-Fuentes M., Letourneux L., Ruiz-Hernández V., Fernández J.A., Egea-Cortines M. (2018). Diel Pattern of Circadian Clock and Storage Protein Gene Expression in Leaves and during Seed Filling in Cowpea (*Vigna unguiculata*). BMC Plant Biol..

[30] Da Silva H.A.P., Nardeli S.M., Alves-Ferreira M., Simões-Araújo J.L. (2015). Evaluation of Reference Genes for RT-QPCR Normalization in Cowpea under Drought Stress during Biological Nitrogen Fixation. Crop Sci..

[31] Amorim L.L.B., Ferreira-Neto J.R.C., Bezerra-Neto J.P., Pandolfi V., Araújo F.T., Silva Matos M.K., Santos M.G., Kido E.A., Benko-Iseppon A.M. (2018). Cowpea and Abiotic Stresses: Identification of Reference Genes for Transcriptional Profiling by QPCR. Plant Methods.

[32] Dong L., Ravelombola W., Weng Y., Qin J., Bhattarai G., Zia B., Zhou W., Wang Y., Mou B., Shi A. (2019). Seedling Salt Tolerance for above Ground-Related Traits in Cowpea (*Vigna unguiculata* (L.) Walp). Euphytica.

[33] Raggi L., Caproni L., Ciancaleoni S., D’Amato R., Businelli D., Negri V. (2024). Investigating the Genetic Basis of Salt-Tolerance in Common Bean: A Genome-Wide Association Study at the Early Vegetative Stage. Sci. Rep..

[34] Nunes L.R.D.L., Pinheiro P.R., Pinheiro C.L., Lima K.A.P., Dutra A.S. (2019). Germination and Vigour in Seeds of the Cowpea in Response to Salt and Heat Stress. Rev. Caatinga.

[35] Tavares D.S., Fernandes T.E.K., Rita Y.L., Rocha D.C., Sant’Anna-Santos B.F., Gomes M.P. (2021). Germinative Metabolism and Seedling Growth of Cowpea (*Vigna unguiculata*) under Salt and Osmotic Stress. S. Afr. J. Bot..

[36] Nikolić N., Ghirardelli A., Schiavon M., Masin R. (2023). Effects of the Salinity-Temperature Interaction on Seed Germination and Early Seedling Development: A Comparative Study of Crop and Weed Species. BMC Plant Biol..

[37] Thiam M., Champion A., Diouf D., Ourèye SY M. (2013). NaCl Effects on In Vitro Germination and Growth of Some Senegalese Cowpea (*Vigna unguiculata* (L.) Walp.) Cultivars. ISRN Biotechnol..

[38] Gogile A., Andargie M., Muthuswamy M. (2013). The Response of Some Cowpea (*Vigna unguiculata* (L.) Walp.) Genotypes for Salt Stress during Germination and Seedling Stage. J. Stress Physiol. Biochem..

[39] Jayawardhane J., Goyali J.C., Zafari S., Igamberdiev A.U. (2022). The Response of Cowpea (*Vigna unguiculata*) Plants to Three Abiotic Stresses Applied with Increasing Intensity: Hypoxia, Salinity, and Water Deficit. Metabolites.

[40] Carvalho M., Castro I., Moutinho-Pereira J., Correia C., Egea-Cortines M., Matos M., Rosa E., Carnide V., Lino-Neto T. (2019). Evaluating Stress Responses in Cowpea under Drought Stress. J. Plant Physiol..

[41] Sairam R.K., Srivastava G.C., Agarwal S., Meena R.C. (2005). Differences in Antioxidant Activity in Response to Salinity Stress in Tolerant and Susceptible Wheat Genotypes. Biol. Plant..

[42] Kaur N., Kumar A., Kaur K., Gupta A.K., Singh I. (2014). DPPH Radical Scavenging Activity and Contents of H_2_O_2_, Malondialdehyde and Proline in Determining Salinity Tolerance in Chickpea Seedlings. Indian J. Geo-Mar. Sci..

[43] Sakuma Y., Maruyama K., Osakabe Y., Qin F., Seki M., Shinozaki K., Yamaguchi-Shinozaki K. (2006). Functional Analysis of an Arabidopsis Transcription Factor, *DREB2A*, Involved in Drought-Responsive Gene Expression. Plant Cell.

[44] Luo Z., Szczepanek A., Abdel-Haleem H. (2020). Genome-Wide Association Study (GWAS) Analysis of Camelina Seedling Germination under Salt Stress Condition. Agronomy.

[45] Razzaque S., Elias S.M., Haque T., Biswas S., Jewel G.M.N.A., Rahman S., Weng X., Ismail A.M., Walia H., Juenger T.E. (2019). Gene Expression Analysis Associated with Salt Stress in a Reciprocally Crossed Rice Population. Sci. Rep..

[46] Chan Z., Loescher W., Grumet R. (2013). Transcriptional Variation in Response to Salt Stress in Commonly Used Arabidopsis Thaliana Accessions. Plant Physiol. Biochem..

[47] Bustin S.A., Benes V., Garson J.A., Hellemans J., Huggett J., Kubista M., Mueller R., Nolan T., Pfaffl M.W., Shipley G.L. (2009). The MIQE Guidelines: Minimum Information for Publication of Quantitative Real-Time PCR Experiments. Clin. Chem..

[48] Hammer Ø., Harper D.A.T., Ryan P.D. (2001). Past: Paleontological Statistics Software Package for Education and Data Analysis Past: Paleontological Statistics Software Package for Education and Data Analysis Even a Cursory Glance at the Recent Paleontological Literature Should Convince Anyone Tha. Palaeontol. Electron..

